# Exercise therapy and patient education versus intra-articular saline injections in the treatment of knee osteoarthritis: an evidence-based protocol for an open-label randomised controlled trial (the DISCO trial)

**DOI:** 10.1186/s13063-020-04952-5

**Published:** 2021-01-06

**Authors:** Elisabeth Bandak, Anders F. Overgaard, Lars Erik Kristensen, Karen Ellegaard, Jørgen Guldberg-Møller, Cecilie Bartholdy, David J. Hunter, Roy D. Altman, Robin Christensen, Henning Bliddal, Marius Henriksen

**Affiliations:** 1 The Parker Institute, Copenhagen University Hospital Bispebjerg-Frederiksberg, Copenhagen, Denmark; 2 Institute of Bone and Joint Research, Kolling Institute of Medical Research, The University of Sydney, Sydney, Australia; 3 Department of Rheumatology, Royal North Shore Hospital, Odense, Australia; 4 Ronald Reagan UCLA Medical Center, Los Angeles, CA USA; 5 Research Unit of Rheumatology, Department of Clinical Research, University of Southern Denmark, Odense University Hospital, Odense, Denmark

**Keywords:** Knee osteoarthritis, Exercise, Education, Intra-articular saline injection, Placebo, Open-label, Randomised controlled trial

## Abstract

**Background:**

Knee osteoarthritis (OA) is a highly prevalent musculoskeletal condition causing pain, physical disability, and reduced quality of life. Exercise and patient education are non-pharmacological interventions for knee OA unanimously recommended as first-line treatments based on extensive research evidence. However, none of the numerous randomised controlled trials of exercise and education for knee OA has used adequate sham/placebo comparison groups because the ‘active’ ingredients are unknown. Designing and executing an adequate and ‘blindable placebo’ version of an exercise and education intervention is impossible. Therefore, using an open-label study design, this trial compares the efficacy of a widely used ‘state-of-art’ exercise and education intervention (Good Life with osteoarthritis in Denmark; GLAD) with presumably inert intra-articular saline injections on improvement in knee pain in patients with knee OA.

**Methods:**

In this open-label randomised trial, we will include 200 patients with radiographically verified OA of the knee and randomly allocate them to one of two interventions: (i) 8 weeks of exercise and education (GLAD) or (ii) Intra-articular injections of 5 ml isotonic saline every second week for a total of 4 injections. Outcomes are taken at baseline, after 8 weeks of treatment (week 9; primary endpoint) and after an additional 4 weeks of follow-up (week 12). The primary outcome is change from baseline in the Knee Injury and Osteoarthritis Outcome Score questionnaire (KOOS) pain subscale score. Secondary outcomes include the Physical function in Activities of Daily Living, Symptoms, and Knee-related Quality of Life subscales of the KOOS, the patients’ global assessment of disease impact, physical performance tests, and presence of knee joint swelling.

**Discussion:**

This current trial compares a presumably active treatment (GLAD) with a presumably inert treatment (IA saline injections). Both study interventions have well-established and anticipated similar effects on knee OA symptoms, but the underlying mechanisms are unknown. The interpretation of the results of this trial will likely be difficult and controversial but will contribute to a better understanding of the bias introduced in the effect estimation of classically unblindable exercise and education interventions for knee OA.

**Trial registration:**

www.ClinicalTrials.govNCT03843931. Prospectively registered on 18 February 2019.

## Background

Knee osteoarthritis (OA) is a highly prevalent musculoskeletal condition mainly affecting older people, causing pain, physical disability, and reduced quality of life. As there is no cure, it imposes a considerable current and future economic burden on the health care system.

Exercise and patient education are non-pharmacological interventions for knee OA unanimously recommended as primary management strategies by leading international organisations and authorities [1–4]. These recommendations are based on extensive research documenting that exercise and education are superior to no-attention control groups. However, none of the numerous randomised controlled trials of exercise and education for knee OA [1–5] has used adequately designed placebo comparison groups because how the underlying mechanism of exercise and education programmes works on symptoms (the ‘active ingredient’) is unknown. This obstructs the design and delivery of ‘inactive’ versions of the interventions as placebos. Furthermore, frequent and lengthy contact with a physiotherapist is standard in exercise and education programmes [6], which, according to current theories, can result in beneficial effects by itself [7, 8]. This is referred to as ‘unspecific’, ‘contextual’, or ‘placebo’ effects. Hence, a significant proportion of the benefits of exercise and education on knee OA symptoms is likely attributable to these types of effects, which can bias the ‘isolated’ effect estimates of exercise and education interventions.

A commonly used placebo-comparator in trials of intra-articular (IA) treatments of knee OA is saline injections [9]. While saline is recognised as a pharmacologically inert agent, a systematic review and meta-analysis concluded somewhat controversially that IA saline injection used as a placebo-comparator in clinical trials for knee OA provides substantial—and clinically relevant—pain relief [9]. The mechanism by which IA saline works on symptoms is—like in the case of exercise—not fully understood, but the considerable clinical effect of saline injections is in line with the current theories that the ‘invasiveness’ of a procedure is a determinant for the magnitude of placebo effects [8].

The ‘active ingredients’ in exercise and education are not known and thereby impossible to simulate and deliver in an inactive version. However, by comparing exercise and education to a presumably inactive treatment with well-established (placebo) effects in an open-label study design, some of the inbuilt challenges that increase risk of biased effects estimation may be mitigated.

In summary, this trial has been designed to compare the effects of an exercise and education programme with IA saline in improving knee pain in patients with knee OA.

### Evidence-based research in support of this trial

No previous trials have compared exercise therapy and education with IA saline. To inform the rationale and design of this trial, we did a meta-epidemiological study aiming at giving a tentative estimate of the comparative effectiveness of exercise therapy and IA saline injections for knee OA pain. From a systematic review of IA saline [9], we extracted data on the short term (≤ 3 months) effectiveness of IA saline for knee OA pain, whereas estimates of short-term effects of exercise were derived from the latest version of the Cochrane systematic review of exercise for knee OA pain [6]. Only data from the IA saline arms or the exercise arms of the RCTs were used. Following the methods presented in the review of IA saline [9], we estimated the total response to IA saline and exercise, respectively, by comparing the effects to simulated matched groups. These simulated groups were given average changes from baseline set to zero with the same dispersion (standard deviation) and a number of observations as in the corresponding exercise/IA saline arms in the same trial. Using these simulated matched groups, we calculated modified standardised mean differences (SMDs) with 95% confidence intervals (95% CIs) for each study. For each intervention (exercise and IA saline injection, respectively), the individual SMDs were pooled using the inverse variance method based on a standard random-effects model. The difference between the two combined estimates of the effectiveness of exercise and IA saline for knee OA pain was calculated and tested as a test for interaction (with a corresponding *z*-test comparing the subgroups) [9].

**Fig. 1:**
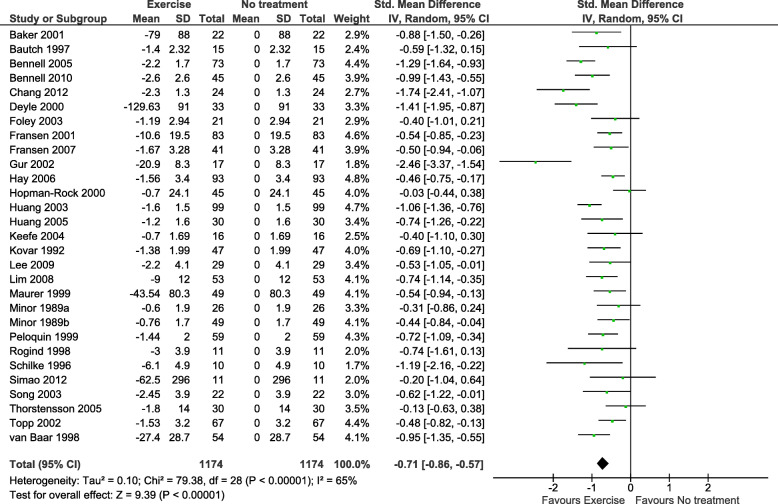
Standardised mean differences of short-term pain changes from baseline comparing exercise vs. no treatment (data modified from Fransen et al. [6])

**Fig. 2:**
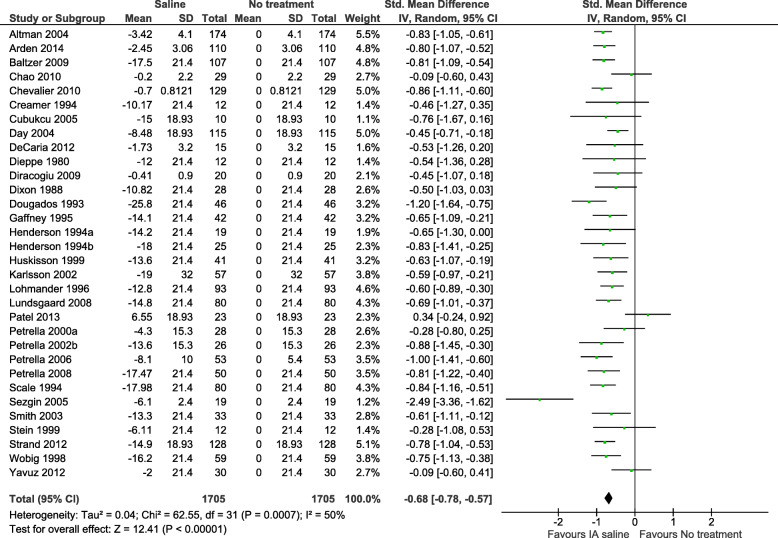
Standardised mean differences of short-term pain changes from baseline comparing IA saline vs. no treatment (data from Altman et al. [9])

Sixty trials were identified (28 exercise; 32 IA saline), with 2879 randomised participants (1705 IA saline, 1174 exercise) in 61 comparisons (29 exercise; 32 IA saline). The combined effect size (SMD) of exercise on knee OA pain assessed between 6 and 16 weeks from baseline corresponded to − 0.71 SMD (95% CI − 0.86 to − 0.57; *P* < 0.00001; *I*^2^ = 65%; Fig. 1). The effect size of IA saline on knee OA pain at the short term (≤ 3 months) was − 0.68 SMD (95% CI − 0.78 to − 0.57; *P* = 0.0007; *I*^2^ = 50%; Fig. 2).

In an indirect comparison of these meta-analysis estimates using previously described methods [9], there was no statistically significant difference between exercise and IA saline (difference: − 0.03 SMD [95% CI − 0.21 to 0.15; *P* = 0.74]) and judged by the precision of the estimate (95% CI) a type-2 error is unlikely (recalling that an absolute effect size of 0.20 would only be considered a small clinical effect) [10]. This indirect comparison indicates that for knee OA pain, the total treatment response from exercise and IA saline is at least comparable, if not similar.

Given the proven benefits of exercise and education for knee OA, yet without adequate placebo-controlled trials in support, we consider it relevant to employ a randomised controlled trial comparing exercise and education with the commonly used placebo comparator IA saline in an open-label study design. The existing evidence provides a clear-cut ethical and scientific justification for the trial described in this protocol.

## Methods/design

### Hypotheses and objectives

The investigators involved in this trial consist of representatives of several professions, including rheumatology, physiotherapy, and biostatistics. As a reflection of the somewhat controversial study aim, the investigators did not necessarily agree on a pre-specified hypothesis. Thus, it was decided to agree to disagree. To map the disagreement each of the involved conceivers, investigators and patient research partners were asked to cast their vote on one of three possible hypotheses:

1. Exercise and education is superior to saline injections on knee OA pain;
2. Saline injections are superior to exercise/education on knee OA pain; or
3. The two study treatments are comparable on knee OA pain.

The outcome of the balloting is given in Table 1 and illustrates the balanced disagreements. As a result, we agreed to work without a prespecified hypothesis and have designed the study to, with reasonable confidence, be able to test the three hypotheses. As equivalence rarely can be claimed from superiority studies, but superiority may be claimed from equivalence studies, we have chosen an equivalence design for this trial, recognising that this is not the main hypothesis.

**Table 1:** Results of the balloting among the trial conceivers, investigators, and patient partners regarding their belief in the outcome of the trial. *Patient partner #1 and #2 prefer to be unnamed in this publication

Trial conceivers and investigators	GLAD superior	Equivalence	IA saline superior
Marius Henriksen	●		
Elisabeth Bandak	●		
Anders F Overgaard		●	
Lars Erik Kristensen			●
Karen Ellegaard		●	
Jørgen Guldberg-Møller			●
Cecilie Bartholdy	●		
David Hunter		●	
Roy D Altman		●	
Robin Christensen			●
Henning Bliddal			●
**Patient partners**			
Patient partner #1*		●	
Patient partner #2*	●		

Accordingly, the primary objective of this trial is to assess efficacy equivalence between an education and exercise programme vs. repeated intra-articular saline injections, on changes in knee pain in individuals with knee OA.

### Patient involvement

Two patient research partners were involved in the process of designing and preparing the study protocol and have acknowledged the protocol in its current form. The patient research partners have acknowledged the idea and purpose of the study and participated in discussions of ethics, design, choice of outcomes, relevance, and feasibility of the investigational programme. They have revised all patient information prior to submission to the authorities. Before their decision of participation, they received a written and oral task description that clarified their roles and expected contributions. The patient partners will be invited to the future interpretation of the results of the trial. The patient partners work voluntarily and will be offered co-authorship of trial-related publications according to the recommendations from the International Committee of Medical Journal Editors criteria (both have declined co-authorship of the present publication).

### Design and setting

**Fig. 3:**
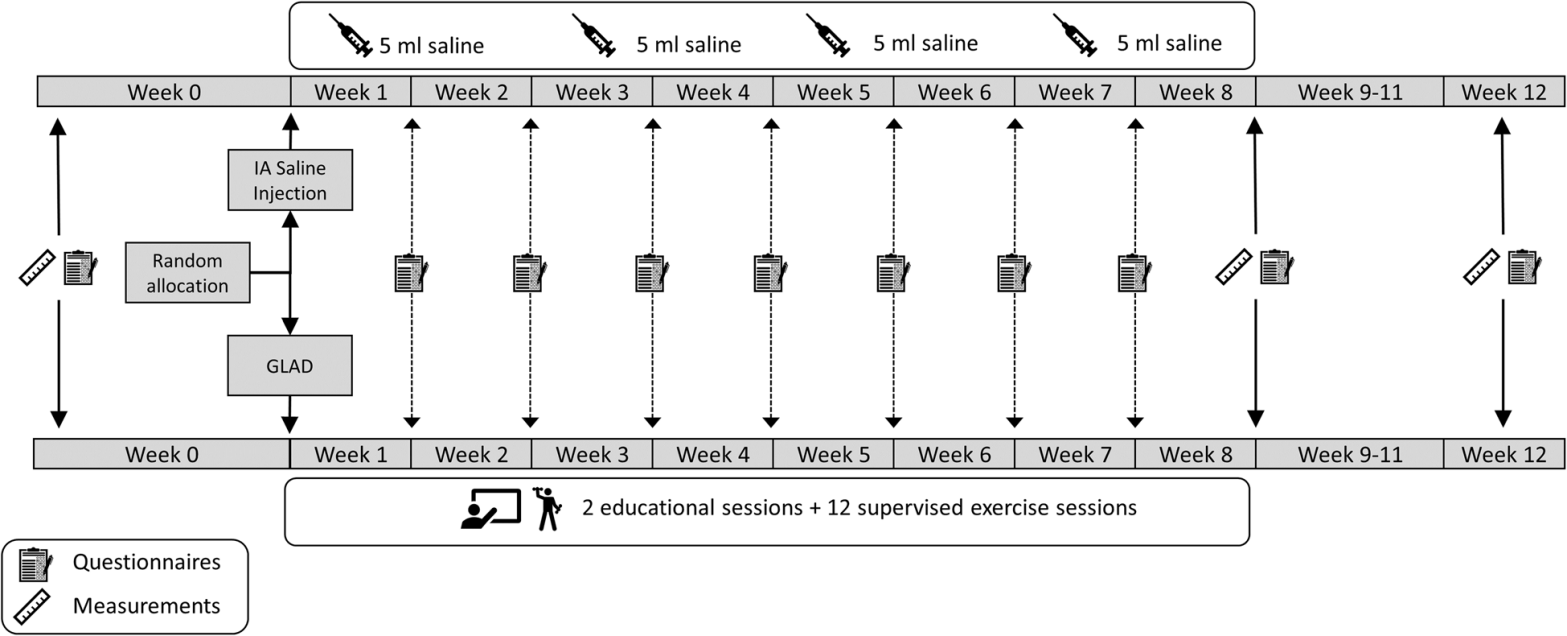
Graphical illustration of the DISCO trial design

This DISCO trial (*D*irect comparison of *I*ntra-articular *S*aline injections with an education plus exercise programme for treatment of knee osteoarthritis symptoms: a randomised, open-label, *CO*ntrolled, evidence-based trial) is designed as a randomised, open-label equivalence trial with two parallel groups and a primary endpoint of change from baseline in knee OA pain at the end of intervention (week 9). The design is illustrated graphically in Fig. 3.

Reporting of the protocol follows the *Standard Protocol Items: Recommendations for Interventional Trials* (SPIRIT) statement [11], the *Template for Intervention Description and Replication* (TiDieR checklist) [12], and the *Consensus on Exercise Reporting Template* (CERT) checklist [13] (checklists in supplementary file 1). Before inclusion of the first participant, the trial was approved by the health research ethics committee of the Capital Region of Denmark (H-19012472), the Danish Health Authority, and registered with ClinicalTrials.gov (NCT03843931) and the European Clinical Trials Database (EudraCT number: 2019-000809-71).

Participants will attend baseline, 9- and 12-week follow-up appointments at the outpatient clinic, whereas links to questionnaires used will be emailed via REDCap (Vanderbilt University, Nashville, TN, USA) to the participants weekly from week 1 to 8. Randomisation was be performed as with stratification, with varying block sizes with a 1:1 allocation (see below for details).

### Selection and allocation of participants

Participants are recruited from the OA outpatients clinic at Bispebjerg-Frederiksberg Hospital in Copenhagen, Denmark, at which all clinical assessments and treatments are provided. Recruitment will be boosted through advertisements in local newspapers. A trained rheumatologist will, upon oral and written information and signed informed consent from potential trial participants, assess eligibility criteria.

### Inclusion criteria

Individuals are eligible for trial participation if the following criteria are met:

1. Age ≥ 50 years
2. Body mass index (BMI) ≤ 35
3. A clinical diagnosis of tibiofemoral OA in the target knee according to the American College of Rheumatology [14]
4. Average knee pain in the last week during weight-bearing activities of at least 4 on a 0 to 10 points scale (0 = no pain; 10 = worst possible pain)
5. Verification of clinical diagnosis by definite tibiofemoral OA on posterior-anterior weight-bearing semi-flexed knee radiographs with severity equivalent to Kellgren and Lawrence (K/L) grade 2 or more

### Exclusion criteria

A potential participant will be excluded from trial participation if any of the following criteria are met:

1. Intra-articular treatments of any either knee within 3 months of the baseline visit
2. Scheduled surgery during study participation
3. Knee joint fluid aspiration within 3 months of the baseline visit
4. Participation in exercise therapy within 3 months of the baseline visit
5. Evidence of other inflammatory joint diseases (e.g. rheumatoid arthritis or gout)
6. History of target knee surgery within 12 months of the baseline visit
7. History of arthroplasty in the study knee
8. Current use of oral glucocorticoids
9. Current use of synthetic or non-synthetic opioids
10. Any condition that precludes participation in exercise
11. Contraindications to IA injections, such as wounds or skin rash over the injection site
12. Contraindications to exercise
13. Planning to start other treatment for knee OA in the trial participation period
14. Regional pain syndromes or generalised pain syndromes
15. Lumbar or cervical nerve root compression syndromes
16. Pregnancy or insufficient anti-conception therapy for fertile females
17. Any other condition or impairment that, in the opinion of the investigator, makes a potential participant unsuitable for participation or which may obstruct participation, such as uncontrolled diabetes, psychiatric disorders, or drug dependency.

### Selection of the study knee

At inclusion, a study knee must be selected, which will be subject to all subsequent assessment (except for systemic or generic evaluations):

- The study knee will be defined as the symptomatic knee with a diagnosis of OA
- If both knees are eligible, the more painful knee will be selected (by the participant)
- If both knees shave equivalent pain, the knee with the greater K/L grade will be chosen
- If both pain and K/L grades are equivalent, the study knee will be randomly assigned (coin flip).

### Allocation of participants, sequence generation and blinding

The trial biostatistician, who is not actively involved in the conduct of the trial, developed the randomisation list (i.e. sequence generation) for allocation of participants to one of the two treatment arms. The randomisation list was generated before inclusion of participants. The randomisation list was computer-generated based on permuted random blocks of variable size (2 to 6 in each block). The allocation ratio is 1:1 stratified for the following four baseline conditions (i.e. 16 mutually independent randomised sequences):

- BMI ≥ 30 kg/m^2^ (yes/no)
- Swollen study knee (present/absent upon palpation)
- Evidence of bilateral tibiofemoral OA defined as K/L grade of at least 2 in both knees from bilateral radiographs
- Positive answer (‘yes’) to the question ‘When you were in your 20’ies did you participate in sports activities for at least 1 hour, 2 times or more per week’?

When a participant is included, and the baseline assessments have been completed, the participant identifier is coupled to one of the treatment arms (depending on stratum) when the including investigator clicks on the ‘randomisation button’, appearing in the electronic case report form system used in the study.

As this is an ‘open-label’ trial, neither the health professionals delivering the interventions nor the participants will be blinded to treatment allocation after randomisation. Outcome assessors will be blinded to treatment allocation where possible. This is of utmost importance, and participants are requested not to disclose their allocation when outcomes are assessed.

### Trial treatments

#### Exercise and education programme

Each participant randomised to the exercise and education programme will be offered participation in the *Good Life with osteoArthritis in Denmark* (GLAD) programme [15]. The GLAD programme is a successful Danish initiative implementing exercise and education according to the international recommendations and was initiated in January 2013. The programme aims at facilitating evidence-based care of patients with OA. The components of the GLAD programme are patient education and exercise therapy delivered by GLAD certified physiotherapists [15]. The GLAD programme is currently available for patients in Denmark, Switzerland, Canada, China, and Australia.

The GLAD programme is an 8-week programme that consists of education and supervised exercise [15]. In this trial, GLAD certified physiotherapists will deliver the GLAD education and exercise intervention. In the clinical application of the GLAD programme, additional treatments (e.g. manual therapy or electrotherapy) are permitted; however, in this trial, no such additions are permitted as this would preclude isolation of the effects of exercise/education.

In this trial, two educational sessions are offered focusing on providing knowledge of knee OA and various treatment options to the participants, with a particular focus on exercise and its benefits. Furthermore, advice about self-management is given. In the original outline of the GLAD programme [15], a third session is provided, in which an expert patient presents his or her experiences with the programme. In this trial, the third session will not be provided. However, to accommodate the personal experience part of the educational programme the physiotherapist encourages new participants in the trial to talk to one of the experienced participants about his/her experiences, effects, and impact so far through the programme.

The educational sessions last about 1.5 h, each with a focus on engaging the participants actively and allowing for the sharing of experiences with each other. The educational sessions take place in the department of physiotherapy at Frederiksberg Hospital. The latest version of the standardised GLAD educational material (PowerPoint presentation slides) will be used. However, due to copyright, the founders of GLAD (copyright and trademark holders) have rejected our request to append the slides to this manuscript (personal communication). A description of the educational sessions is available in supplementary file 2.

The exercise part of the GLAD intervention consists of 12 supervised exercise sessions of approximately 60 min each. The sessions are group-based, and the exercise programme is the so-called NEuroMuscular Exercise programme (NEMEX) [15–20]. The supervised exercise sessions take place in the exercise facilities in the department of physiotherapy at Frederiksberg Hospital. A group will consist of up to 12 participants with running uptake. The founders of GLAD (copyright and trademark holders) have declined our request to append the original exercise programme description that is used by GLAD certified physiotherapists. We have been granted permission to reproduce the exercise programme description with our own illustrations and photographs. A full description of the exercise programme with illustrations is available in supplementary file 2.

In this trial, individual exercise diaries will be filled out by the participants and checked by the physiotherapist to ensure documentation of the actual exercises performed. Further, we will monitor pain at arrival to an exercise session as well as pain at the end of each session on a 0–10 points numerical rating scale. Such exercise documentation and pain monitoring are not parts of the original GLAD programme.

#### Intraarticular saline injections

Each participant randomised to IA saline injection will be offered 4 IA injections of 5 ml sterile, isotonic solution of sodium chloride in sterile water (0.9% = 9 mg/ml) in weeks 1, 3, 5, and 7 after baseline, using ‘no-touch’ technique. The injections will be delivered at the osteoarthritis outpatients clinic at Frederiksberg Hospital. The injections will be carried out with a 21-gauge (38 mm) needle and a 5-ml Luer-lock syringe under ultrasound guidance. The injections are delivered by two physiotherapists who have 15 and 7 years of experience, respectively, with ultrasound-guided IA injections and who perform these routinely under the supervision and regulation of a specialist in rheumatology (HB). During ultrasound imaging guidance (Logic E9, General Electrics Medical System with a 15-MHz linear array transducer, Milwaukee, WI, USA), the needle is inserted into the suprapatellar pouch of the study knee in a lateral oblique approach [21]. The procedure is documented in real-time on the ultrasound monitor, ensuring correct deposition of the bolus in the joint cavity. The injections will be conducted under aseptic conditions with standardised skin preparations. No local analgesics are used.

If excessive joint fluid is detected during the ultrasound-guided preparation of the injection, this will be aspirated—if possible—before injection of the saline. If excessive fluid is detected, a 21-gauge (38 mm) needle will be used and the fluid will be aspirated in a 20-ml syringe, a syringe conductor will be used allowing to change to the syringe with saline, and thus, the participant will only get one injection even though fluid is aspirated from the anterior part of the joint. If excessive fluid in the posterior part of knee (Bakers cyst) is seen, this may be aspirated if deemed clinically necessary. The volume of the aspirated fluid both anteriorly and posteriorly will be recorded. We will monitor knee pain at arrival to an injection session as well as knee pain at the end of each session on a 0–10 points numerical rating scale.

#### Concomitant treatments

Analgesics in the form of acetaminophen (paracetamol), NSAIDs, and acetylsalicylic acid are allowed and must be documented in the CRF. On the days that participants are scheduled to be seen in the clinic, no analgesics should be used within 48 h of the participant’s visit to the clinic. Doses of analgesics can be tapered. Initiation of opioids or oral glucocorticoids is not allowed during the study participation. Intra-articular injections to the lower extremities and aspiration of joint fluid from either knee, except those described in this protocol, are not allowed during the study participation. Initiation of non-pharmacological treatments for the lower extremity, except those described in this protocol, is not allowed during the study participation. Surgical treatments to the lower extremity are not allowed during the study participation. Concomitant treatments do not necessarily imply discontinuation of the participant from the study but are considered a violation of this protocol.

### Outcomes

The assessment schedule is presented in Table 2.

**Table 2:**
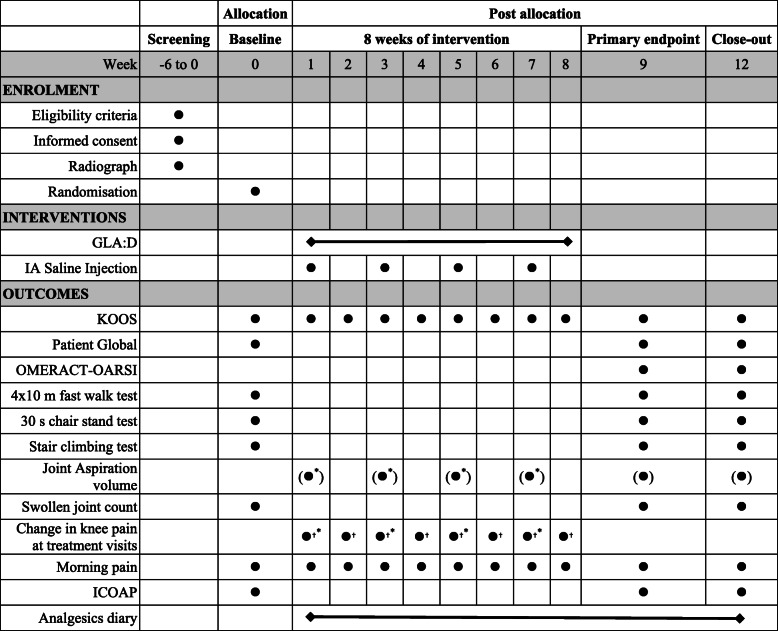
Schedule of enrolment, interventions and assessments

Bullets in parentheses ( ) indicate ‘if possible’ and pertain to joint fluid aspiration that can only be done if excess joint fluid is present

^*^IA Saline group

^†^GLA:D group

*KOOS* Knee injury and Osteoarthritis Outcome Score

*OMERACT-OARSI* Outcome Measures in Rheumatology-Osteoarthritis Research Society International responder criteria

*ICOAP* Intermittent and Constant Osteoarthritis Pain questionnaire

### Primary outcome

The primary outcome is assessed at week 9 as change from baseline in the Knee Injury and Osteoarthritis Outcome Score (KOOS) pain subscale.

The KOOS is a disease-specific instrument designed to assess the patient’s opinion about their knee and associated problems. It consists of 42 items covering five domains: Pain (9 items), Symptoms (7 items), Physical function in Activities of Daily Living (17 items), Sports and Recreation (5 items), and Knee-related quality of life (4 items). The KOOS uses a 5-point Likert scale scoring system (ranging from 0 [least severe] to 4 [most severe]). A normalised 0–100 score is calculated for each subscale with 100 indicating no symptoms and functional impairment and 0 indicating extreme symptoms and functional impairment. The KOOS has been validated for short- and long-term follow-up studies of knee injury [22, 23] and is considered reliable and responsive [24].

### Secondary outcomes

Secondary outcomes are assessed at weeks 9 and 12 and include changes from baseline in (i) the remaining subscales of the KOOS, (ii) The participant’s global assessment of impact of OA, (iii) 4 × 10 m fast walk test, (iv) the 30 s chair stand test; (v) stair climbing test, and (vi) the number of treatment responders according to the OMERACT-OARSI response criteria.

The participant’s global assessment (PGA) of impact of osteoarthritis will be assessed on a 100 mm visual analogue scale (VAS) relating to the degree of the participant’s perceived impact of their knee OA on their overall life, with anchors: 0 = ‘no impact’ and 100 = ‘worst imaginable impact’.

According to the recommended performance-based test to assess physical function in people diagnosed with knee OA [25], we assess the 4 × 10 m fast walk test (40mFWT), the 30 s chair stand test (30sCST), and a stair climbing test (SCT). The 40mFWT is a measure of walking speed over short distances with changing direction. The participant is asked to walk as quickly but as safely as possible to a mark 10 m away, return, and repeat for a total distance of 40 m. Time of one trial, with turn times excluded, is recorded and expressed as speed in m/s by dividing distance (40 m) by time (s). The 30sCST is a physical performance test that quantifies how many sit-to-stand movements an individual can perform within 30 s. From the sitting position in the middle of a seat with feet shoulder-width apart, flat on the floor, arms crossed at the chest, the participant is asked to stand entirely up, then sit entirely back down, repeatedly for 30 s. The total number of complete chair stands (up and down represents one stand) is counted. There is only one trial. If a full stand is completed at 30 s, then this is counted in the total. The same chair is used at all assessments. The SCT is a physical performance test that quantifies how fast an individual can ascend and descend a flight of stairs in a usual manner. The SCT is a measure of balance during functional activities and lower extremity function and strength. The participant is asked to ascend and descend a flight of stairs in a usual and safe manner, as quickly as possible. Use of any walking aid and handrail is permitted and recorded. Total time to ascend and descend steps for one trial is recorded in seconds. The same flight of stairs is used at all assessments.

Per the OMERACT-OARSI criteria [26], a patient is classified as a positive responder if at least one of the following two conditions is observed at the post-baseline assessment:

- In either KOOS pain subscale or KOOS function in activities of the daily living subscale, a high improvement in the subscale, where high improvement in a subscale is achieved if there is both a > 50% improvement from baseline and an absolute change from baseline of > 20 normalised units (0–100 scale), OR
- At least two of the following:
  1. Improvement in KOOS pain defined as > 20% improvement from baseline *and* an absolute change from baseline of > 10 normalised KOOS pain points (0–100 scale)
  2. Improvement in KOOS function in activities of daily living defined as > 20% improvement from baseline *and* an absolute change from baseline of > 10 normalised KOOS function points (0–100 scale)
  3. Improvement in PGA defined as > 20% improvement from baseline *and* an absolute change from baseline of > 10 mm (0–100 scale)

### Other outcomes

At baseline, the 9- and 12-weeks assessments, an investigator (medical doctor) will examine the target knee and record if it is swollen or not based on the presence of palpable effusion. The outcome of the examination will be recorded as a dichotomous score (present/absent).

To assess aspects of the knee OA pain experience not captured by other instruments used in the trial, we apply the Intermittent and Constant Osteoarthritis Pain questionnaire (ICOAP) [21]. The ICOAP is a diagnosis-specific questionnaire designed to assess the pain experience within the last week among people suffering from knee and hip OA. The questionnaire is divided into two domains, a 5-item scale for constant pain and a 6-item scale for intermittent pain (so-called pain that comes and goes). Each domain captures pain intensity as well as related distress and the impact of OA pain on quality of life. For each of these pain types, single items assess pain intensity, the effect on sleep, impact on the quality of life, the extent to which the pain ‘*frustrates or annoys*’, and the extent to which it ‘worries or upsets’. For pain that comes and goes, two additional items ask respondents to report the frequency of pain and the degree to which the pain could be predicted. All items are scored on a 5 point Likert scale—for questions asking about intensity, response options are 0: ‘not at all’ to 4: ‘extremely’, while those that asked about frequency has the following response options: 0 ‘never’ to 4 ‘very often’. A score is separately produced for the constant pain subscale (range 0–20) and the intermittent pain subscale (range 0–24), and total pain (range 0–44). Normalised scores for the two subscales and the total pain score, from 0 (no pain) to 100 (extreme pain), are calculated. Further, the participants’ average morning pain during the last week is recorded at baseline, weeks 9 and 12 using a 100-mm visual analogue scale (VAS) with anchors: 0 = ‘no pain’ and 100 = ‘worst imaginable pain’.

At the 9- and 12-weeks follow-up assessment, presence of excess joint fluid will be visualised during an ultrasound examination and, if possible, aspirated by inserting a needle into the suprapatellar pouch or posterior to the posterior bursa (Bakers Cyst) (under ultrasound guidance). The volume (in ml) of the aspirated fluid will be recorded.

During the intervention period of the trial (week 1 to 8), we will assess the KOOS questionnaire and the average morning knee pain every week via online questionnaires with individual links emailed to the participants via REDCap (Vanderbilt University, Nashville, TN, USA). The frequent emails may also promote participant retention.

To assess if there are immediate responses to the investigational treatments, we will monitor the current target knee pain at each treatment visit. At arrival to each treatment session (IA saline injection, exercise and education), we will ask the participants to assess their current knee pain in the target knee a 0–10 points numerical rating scale, with 0 representing ‘no pain’ and 10 representing ‘worst imaginable pain’. The assessment is repeated when the participants leave the session. Finally, the participants’ use of over-the-counter paracetamol and ibuprofen are recorded using a diary that is handed out to the participants at baseline and collected at the 9- and 12-weeks follow-up assessments.

### Adverse events

The incidence of adverse events with exercise therapy on knee OA is largely unknown as it is sporadically reported [27]. However, one study reported an increased incidence of palpable effusion after participation in a 3-month exercise programme [16]. A pooled analysis of two of our previous studies [28, 29] that use the same exercise protocol showed that 28% of participants were temporarily referred to a modified programme due to symptomatic exacerbations [30]. The referrals were predominantly occurring in the initial phase of the programme, which could reflect that conditioning is taking place, and that initial symptomatic exacerbation is a natural part of the treatment. Nevertheless, unwanted reactions can occur, particularly in case of ‘overdoing’ the exercises. Such a reaction can include muscle soreness, fatigue, symptomatic exacerbations, and joint effusions. The participants will be informed of this risk and post-procedure care as well as signs and symptoms that require medical attention that will be provided by the investigators if necessary. In case of adverse events, these will be recorded, and the participants will be referred to an appointment with an investigator (medical doctor) for resolution of any adverse events.

With IA saline injections, joint sepsis is a primary concern. However, while its incidence is difficult to measure with certainty, it is very infrequent. A 1999 estimate from a retrospective survey in France suggests an incidence rate of 13 per million injections [31]. Though adverse reactions to an isotonic physiological fluid are not expected, adverse events could occur because of the technique of administration or contamination of the fluid. These could result in a febrile response, local tenderness, abscess, tissue necrosis, thrombosis, or infection at the site of the injection. The likely incidence of such events is extremely low, which also corresponds to the clinical experience in our department where standard operating procedures are used to minimise the risks. Nevertheless, participants will be informed of this risk and post-procedure care as well as signs and symptoms that require medical attention that will be provided by the investigators if necessary. In case of adverse events, these will be recorded, and the participants will be referred to an appointment with an investigator (medical doctor) for resolution of any adverse events.

The trial may be terminated at any time for reasons that include, but are not restricted to, the incidence of events in this or other studies that indicate a potential health hazard to participants.

### Data management and quality control

The collection, preservation, and dissemination of the clinical data are specified in the full trial protocol and abide by the standard requirements for Good Clinical Practice (GCP) compliant data management in clinical trials. The source data and documents, eCRF, protocol and amendments, drug accountability forms, correspondence, patient identification list, informed consent forms, and other essential GCP documents will be retained for at least 10 years after the study is completed at the study site. All data collected during this project will be managed and quality certified by the Parker Institutes data management team. This team is responsible for ensuring data completeness and accuracy as well as source data verification. The team is also responsible for ensuring operations of a secure database established for the collection of clinical data collected via the eCRF platform through a secure connection. An external monitoring committee (The Good Clinical Practice Unit at Copenhagen University Hospitals) will oversee the trial. The monitor has visited the trial site before trial commencement and after that, visits the trial site regularly. The monitor will check trial procedures, including safety assessments, drug handling, data recording and complete source data verification procedures, and participant confidentiality. All data obtained during the study will be documented in the individual eCRFs. Reasons for any missing data will be noted in the database, and logging and tracking of data changes will be documented. All the investigators will have full access to the dataset.

### Power and sample size considerations

As stated previously, we agree not to agree on the a priori hypothesis. Thus, to be able to design a rigorous study, we have chosen to pursue an equivalence study design per se. The sample size needed for this study is 200 based on the following sample size calculations based on the primary outcome: change in KOOS pain from randomisation to week 9 (end of treatment).

#### Equivalence claim

The sample size is calculated to test the equivalence of GLAD versus IA saline injections in the assessment of change in the KOOS pain subscale 9 weeks after randomisation: In a two one-sided tests analysis for additive equivalence of two-sample normal means with equivalence margins of − 8 and 8 KOOS pain subscale points (0–100 scale) for the mean difference and a statistical significance level of 0.05 (two one-sided significance levels of 0.025), assuming a mean difference of 0 KOOS pain subscale points and a common standard deviation of 15 KOOS pain subscale points, a total sample size of 154 assuming a balanced design (1:1) is required to obtain a statistical power of 90%. To account for a possible dropout rate (of up to 23%), it was decided to aim for 200 patients in total. If, however, recruitment difficulties arise, a total sample size of 122 (assuming a balanced design and dropout rate) is required to obtain a statistical power of 80%.

#### Superiority claim

A sample size of 154 (as illustrated above) will have a power of 82.1% to detect a mean difference of 7 KOOS-pain points assuming a balanced design and a common standard deviation of 15 KOOS-pain points using a two-sample pooled t-test of a normal mean difference with a two-sided significance level of 0.05. The planned sample size of 200 in total accounts for a dropout rate of up to 23% and provides statistical power of 80.4% to detect a 6 KOOS-pain points group difference (0–100 scale) using a two-sample pooled *t* test of a normal mean difference with a two-sided significance level of 0.05 (*P* < 0.05), assuming a common standard deviation of 15 KOOS-pain points (corresponding to a small effect size of 0.4). If, however, recruitment difficulties arise, even a total sample size of 114 corresponds to a statistical power of 80.6% to detect a mean difference of 8 KOOS-pain points assuming a balanced design (approximately 57 participants in each group) and a common standard deviation of 15 KOOS-pain points using a two-sample pooled *t* test of a normal mean difference with a two-sided significance level of 0.05.

### Analysis populations

The intention-to-treat (ITT) population will consist of all randomised patients irrespective of whether the patient received study intervention or the patient’s compliance with the study protocol, in the treatment group to which the participant was assigned at randomisation. A patient will be considered randomised as soon as a treatment is assigned according to the allocation sequence (i.e. when a virtual envelope is opened).

The per-protocol (PP) subset population is defined as participants that adhere to this protocol, defined by the following criteria to the two intervention groups:

GLAD:

- ▪ Have a baseline measurement, AND
- ▪ Have a 9-week measurement, AND
- ▪ Have attended at least 1 of the educational sessions, AND
- ▪ Have attended at least 8 of the scheduled exercise sessions, AND
- ▪ Comply with the rules for concomitant treatments, AND
- ▪ Have no major protocol violations

IA saline:

- ▪ Have a baseline measurement, AND
- ▪ Have a 9-week measurement, AND
- ▪ Have received at least 3 and maximum 4 of the scheduled injections, AND
- ▪ Comply with the rules for concomitant treatments as described above, AND
- ▪ Have no major protocol violations

### Statistical analyses

A statistical analysis plan that describes the details of the planned statistical analyses will be produced by the biostatisticians and published at the Parker Institute’s website (www.parkerinst.dk) before the last participant’s last visit.

Statistical analyses will be performed on the ITT and the PP populations. For the ITT population analysis, missing data will be handled implicitly by using repeated measurements mixed linear models [32]. For the PP population analysis, missing data will per definition not be a problem. Continuous outcomes will be analysed using repeated measures mixed linear models, including participants as random effects, with a fixed effect factor for group and week and the corresponding interaction (i.e. group × week), adjusted for baseline values and the categorical variables used for the stratified randomisation.

To assess the adequacy of the linear models describing the observed data—and checking assumptions for the systematic and the random parts of the models—we will investigate the model features via the predicted values and the residuals; that is, the residuals must be normally distributed (around 0) and be independent of the predicted values. Results will be expressed based on the least squares mean estimates of the group differences in the changes from baseline, with 95% CIs to represent the precision of the estimates.

Equivalence will be claimed if the computed 95% CI of the estimated group difference in the change from baseline in the KOOS pain subscale does not include ± 8 KOOS points in the PP population. Superiority will be claimed if the computed 95% CI of the estimated group difference in the change from baseline in the KOOS pain subscale does not include 0 in the ITT population. All 95% confidence intervals (and corresponding *P* values) will be two-sided. We will not apply explicit adjustments for multiplicity, rather we will analyse the secondary outcome measures in a prioritised order.

### Dissemination

The DISCO trial is planned to be reported in scientific peer-reviewed journals. The results will also be presented at relevant scientific conferences and symposiums. Trial participants will be informed of the results at a participant symposium. All contributors to the study will be offered authorship if they fulfil the International Committee of Medical Journal Editors (ICMJE) recommendations for authorship. No professional writers will be used.

## Discussion

This paper describes the methodology of the ongoing, open-label DISCO trial, designed to compare a widely used education and exercise therapy programme (GLAD) with IA saline injection, a common placebo comparator in clinical trials.

In pharmacological trials, participants and care providers are typically blinded to placebo and active interventions. This is a challenge in trials of exercise and education, in part because the ‘active ingredients’ of exercise and education are unknown and thereby impossible to simulate and deliver in an inactive version. The lack of adequate placebo-controlled studies leads to biased effect estimates. However, by applying an open-label study design, and comparing exercise and education to a common placebo treatment with well-established effects (IA saline), some of the inbuilt challenges that increase the risk of bias may be mitigated.

In OA, previous studies have used masked placebos as comparators against multi-modal physiotherapy (education, advice, massage, taping, manual mobilisation, and exercise) for hip [33] and knee [34] OA. The physiotherapy programmes tested were no more effective than the applied placebos. Currently, the TEMPO trial [35] is applying a placebo physiotherapy arm in a 4-armed RCT on meniscal tears in knee OA, in which the participants are blinded, but providers are not. Importantly, the blinding of study participants in these studies implies some degree of deception. We have chosen an open-label study design to avoid the deliberate deception of the participants. Finally, the comparable efficacy of exercise (the main component of GLAD) and IA saline (Figs. 1 and 2) makes the open-label design easier to explain honestly to the participants.

### Culture of the trial

A specific point in this trial is the direct comparison of a presumably active treatment (GLAD) with a presumably inert treatment (IA saline injections), with indirect evidence of similar efficacy. In our experience, potential trial participants often consider inert treatments as ‘losing tickets’ in the ‘randomisation lottery’. We will aim to mitigate this belief by emphasising that both GLAD and saline injections are expected to provide significant improvements in knee OA symptoms and that the underlying mechanisms of neither GLAD nor IA saline injections are understood. Further, we will advertise that both IA saline injections and GLAD are safe. We will try to avoid advertising GLAD to make people think that the IA saline injection is the less important treatment. Likewise, some patients may want a treatment that requires less effort and, therefore, we will also try to avoid advertising IA saline injections as a ‘quick fix’. When potential participants contact the trial, we will explain the trial transparently and clearly explain that we compare IA saline injections and GLAD to explore and understand the effectiveness of both interventions. Further, we will explain that there is some evidence that the benefits of the two interventions are comparable. Thus, both interventions in this trial will be accompanied by positive messages from the trial staff, and the delivery of the interventions will be in a warm and friendly atmosphere, which altogether may help maintaining adherence. Further, we will tell the participants that the ‘active ingredients’ in both interventions are very difficult to describe and involves the sum and/or interaction of many factors.

### Strengths

There are several strengths to the DISCO trial. Firstly, the involvement of patients in developing the protocol ensures a high level of relevance to the target audience. Secondly, the GLAD intervention is applied internationally, which also increases relevance globally. Also, GLAD includes patient education, which is important for successful self-management of knee OA and rheumatic diseases in general and recommended by EULAR [4, 36]. Thirdly, the selection of outcomes was made according to the current OMERACT-OARSI core outcome set for knee OA [37] with the inclusion of suggestions from our patient partners. Finally, a unique yet unconventional aspect of the DISCO trial is that both interventions (GLAD and IA saline injections) are delivered by physiotherapists. This prevents a subsequent discussion of possible group differences occurring due to different professions delivering interventions (i.e. medical doctor vs. physiotherapist).

### Limitations

There are limitations and unaddressed questions in our trial. One of these is whether GLAD or IA saline generates objectively measurable changes in disease-specific outcomes, such as cartilage loss. However, in studies on OA using a concealed placebo, there is no evidence of structural effects of placebo [8]. In clinical trials in general, the placebo effects are known to be amplified by subjective reporting of outcomes, the invasiveness of an intervention and frequent attention from caring health professionals [8]. These determinants of placebo are all part of the present DISCO trial and may render the interpretation of their relative contribution to effects difficult. Another limitation is the two-group study design. The study could have been further informative if a no-attention control group was added. However, we decided on the two-group comparison based on expected recruiting difficulties arising from the inclusion of a no-attention control group (a true losing ticket in the randomisation lottery). Also, the lack of long-term measures of efficacy may be regarded as a limitation, but we believe that prolonged observation of patients with a chronic disease increases the chance of data pollution, i.e. participants may seek other treatments, which will hamper interpretation of long-term follow-up data. Also, the mechanisms of the trial interventions’ long-term efficacy are even less understood than those of short-term. Further, some of the questionnaires are delivered by email, which may limit the generalisability of these study results to certain populations.

### Trial status

The protocol was first prospectively registered at www.ClinicalTrials.gov (NCT03843931) on February 18, 2019. Recruitment was started on July 30, 2019, and the first participant was included on August 5, 2019. No amendments have been made to the protocol (version 1.5 June 28, 2019) or the registration since recruitment of the first participant. Any future amendments will be communicated together with the results. When this manuscript was submitted for publication (May 19, 2020), a total of 110 participants had been included in the trial. We expect recruitment to be completed in December 2020.

On March 12, 2020, a lockdown of all non-critical activities in the public sector in Denmark was decreed by the Danish government due to the COVID-19 pandemic. This lockdown forced us to suspend the DISCO trial as an urgent safety measure. The suspended trial activities include screening visits, baseline assessments, intervention delivery, and all clinical outcome assessments. Scheduled outcome assessments (weeks 9 and 12) has been conducted as telephone interviews, where patient-reported outcome measures will be collected as well as information about adverse events.

On the date of the suspension, 27 participants were active in the trial. Of these, 15 were allocated to GLAD and 12 to IA saline. For those allocated to GLAD, 13 had started the intervention and 2 had not started the intervention. For those allocated to IA saline, 9 had started the intervention (3 had received the first injection, 2 had received the second injection, 3 had received the third injection, and 1 had received the fourth injection) and 3 had not received the first injection.

On April 20, 2020, some activities at the OA outpatient clinic at Bispebjerg-Frederiksberg Hospital were resumed, including those related to clinical trials.

On April 23, 2020, the suspension of the present trial was lifted, with the only restriction being that planning and execution of the group-based education and exercise interventions will ensure that a maximum of 10 persons (including trial staff) are in the same room, and that a distance between individuals of at least 2 m can be enforced. Use of Personal Protective Equipment (masks, visors etc) is allowed and recommended during trial-related activities.

The trial steering committee has decided to manage the participants whose participation has been affected by the suspension as follows:

- For the participants who had not received their first treatment at the time of the suspension (GLAD *n* = 2; IA saline *n* = 3) the baseline measurements were repeated and treatment was started according to the protocol (with restrictions as described above). The repeated baseline assessments (after allocation) have been recorded as protocol violations.
- For the participants who had begun the investigational treatments in the trial at the time of suspension (GLAD *n* = 13; IA saline *n* = 9) patient-reported outcome measures at weeks 9 and 12 (including primary outcome) were collected via telephone interviews at the scheduled time points. The outcomes that require a clinical visit have not been collected and are defined as missing completely at random for these participants. No treatments were delivered during the suspension and the trial participation for the involved participants has not been extended. These deviations have been recorded as protocol violations.

The COVID-19 pandemic and associated restrictions may have affected the participants both directly and indirectly [38, 39]. While the trial suspension was relatively short-lived, the impact of the pandemic will likely continue after the restart of the trial. Specific handling of missing data due to the COVID-19 pandemic and any assessment of the influence of the COVID-19 pandemic on the study data will be considered and described in the statistical analysis plan if considered relevant.

### Perspectives

The underlying pain-relieving mechanisms of exercise and education in knee OA are largely unknown, but the benefits have been documented in numerous studies [6]. In the current trial, we expect similar benefits of the GLAD programme, but the study is not designed to expand the knowledge about the underlying mechanisms. Likewise, the mechanisms of open-label placebo are far from understood [40], but significant symptomatic improvements have been documented in irritable bowel syndrome [41], chronic low back pain [42], and episodic migraine [43]. In the present trial, we expect beneficial effects of the IA saline, but the study is not designed to expand the knowledge about open-label placebo. Rather, this trial may highlight the importance of contextual factors when estimating treatment responses to exercise and education as well as IA saline in knee OA.

The interpretation of the results of this trial will likely be difficult and perhaps controversial. If the participants who are knowingly receiving placebo (IA saline) experience similar clinical benefits as the participants knowingly receiving GLAD, can we then claim that GLAD is not better than inert placebo or that IA saline is not inert? If GLAD is superior to IA saline, can we then estimate the proportions of placebo and specific effects of GLAD? What if IA saline is superior to GLAD? We may not be able to answer these questions completely with confidence, but the results may inform ongoing and future efforts to discover the mechanisms by which many treatments relieve knee OA pain. Further, the current trial may allow some tentative inferences on the possible use of IA saline as a supplementary treatment in knee OA.

## Supplementary Information

**Additional file 1.** THE DISCO TRIAL SPIRIT 2013 Checklist.

**Additional file 2.** Description of the exercise and education program.

## Data Availability

The full trial protocol, statistical codes, and fully anonymised participant-level datasets can be made available in fully anonymised form upon reasonable request.

## Abbreviations

OA: Osteoarthritis
IA: Intra-articular
SD: Standard deviation
SMD: Standardised mean differences
CI: Confidence interval
KOOS: Knee Injury and Osteoarthritis Outcome Score
SPIRIT: Standard Protocol Items: Recommendations for Interventional Trials
TiDieR: Template for Intervention Description and Replication
BMI: Body mass index
K/L: Kellgren and Lawrence
GLAD: Good Life with osteoArthritis in Denmark
NEMEX: NEuroMuscular Exercise programme
PGA: Patient’s Global Assessment
40mFWT: 4 × 10 m fast walk test
30sCST: The 30 s chair stand test
SCT: Stair climbing test
ICOAP: Intermittent and Constant Osteoarthritis Pain questionnaire
VAS: Visual analogue scale
GCP: Good Clinical Practice
ITT: Intention to treat
PP: Per protocol
