# Supplementary material for: Exercise therapy and patient education versus intra-articular saline injections in the treatment of knee osteoarthritis: an evidence-based protocol for an open-label randomised controlled trial (the DISCO trial)
Source: Trials. 2021 Jan 6;22:18. doi: 10.1186/s13063-020-04952-5 (PMC7787248; doi:10.1186/s13063-020-04952-5)
Supplement: Supplementary file 2 — Additional file 2. Description of the exercise and education program. [file 13063_2020_4952_MOESM2_ESM.pdf]

DESCRIPTION OF THE EDUCATION AND EXERCISE PROGRAM USED IN THE DISCO TRIAL

The program used in this trial is delivered by physiotherapists whose role is to deliver osteoarthritis (OA) care according to clinical guidelines including patient education, practical instructions and supervision in neuromuscular exercise according to the description of the Good Life with osteoArthritis in Denmark (GLAD) program (1). Thus, the program used in this trial follows the original GLAD program and is based on specific GLAD educational material and a GLAD exercise program. However, the founders of GLAD (copyright and trademark holders) have declined our request to use the GLAD educational material and GLAD exercise program descriptions in this publication.

For reporting clarity and completeness and clinical replicability purposes, we have added some further details (2, 3). Where the present program deviates from the originally described GLAD program, this is explicitly stated.

GENERAL LAYOUT OF THE PROGRAM

The GLAD program is delivered by physiotherapists that have been GLAD certified by participation in a GLAD Certification Course. Certified GLAD physiotherapists have access to the educational material and the exercise program.

The core components of the program are 2 educational sessions and a 6-week exercise program with 2 group sessions weekly (1). In this trial the program is delivered at our facility (Department of Physiotherapy at Frederiksberg Hospital). In the description of the original GLAD program the program is designed to be deliverable in several settings, including private clinics, municipality rehabilitation centres, or hospital settings (1, 4).

In the original GLAD concept, a third component is also described: registration of patient data in a national registry. In this trial we do not register the patients or their data in the registry, nor are they asked to submit their data to the registry.

EDUCATION

Two educational sessions are offered focusing on providing knowledge of OA and various treatment options to the participants, with a special focus on exercise and its benefits.

The first session provides knowledge on OA in the knees, hips, and fingers including prevalence, structures affected, risk factors, and symptoms. Furthermore, various treatment and management options are presented including insoles, electrotherapy, acupuncture, and surgery.

The main focus in the second session is exercise as treatment of OA. The session underlines the relevance of being physically active in prevention of lifestyle diseases as coronary heart disease and diabetes. Several examples are given of the beneficial effects of physical activity including positive effect on blood pressure, blood lipids, blood sugar, cardiovascular fitness, muscles, bones, cartilage, weight, pain relief, and mood.

Examples of physical activity are given including activities as walking, sports, dancing, gymnastics, and strength training. The difference of being physically active and exercising is a part of the session. The benefits of exercise in OA are presented containing the following headlines: 'Exercise in OA – why?'; 'Exercise – how?'; and 'Exercise - what?'. Furthermore, separate information on pain management during exercise as well as advice about self-management are a part of the second session.

Each educational session lasts approximately 1.5 hours with a focus on engaging the participants actively and allow sharing of experiences with each other. These education sessions are group-based led by a GLAD certified physiotherapist. A group is typically consisting of 6-12 participants with running uptake resulting in groups with both novice and experienced participants. The latest version of the standardised GLAD educational material (Microsoft power point presentation slides) will be used. Due to copyright, the founders of GLAD (copyright and trademark holders) have declined our request to append the standardised GLAD educational material to this manuscript (personal communication).

In the original GLAD program, a third session is described in which an expert patient is presenting his/her experience with the program (or optionally a reference to an online resource with a video of a previous GLAD participant can be provided). In this clinical trial the third session will not be provided; however, the physiotherapist encourages new participants in the trial to talk to one of the experienced participants in the gym about his/her experiences, effects and impact they have experienced so far through the program.

The educational sessions take place at the Department of Physiotherapy at Frederiksberg Hospital in an educational round-table setup.

EXERCISE

The exercise part of the program lasts for 6 weeks with two exercise sessions per week each lasting approximately 60 minutes. These exercise sessions are group-based at our facility supervised by a GLAD certified physiotherapist. A group is typically consisting of 6-12 participants with running uptake resulting in groups with both novice and experienced participants. All the scheduled exercise sessions are performed at the Department of Physiotherapy at Frederiksberg Hospital.

Each exercise session consists of three parts: a warm-up part, a circuit program part and a cool-down part.

Part 1: The warm-up consists of 10 minutes of ergometer biking. The workload is individually adjusted and may be gradually increased over the course of the program.

Part 2: The circuit part comprises of four exercise 'stations' each with a specific focus: Core stability; alignment of joints; lower extremity muscle strength; and functional exercises. At each 'station' 2-4 exercises are performed. Each exercise should be performed in 2-3 sets of 10-15 repetitions, with rest between sets corresponding to one set. The exercises are performed with both legs, although focus is on the affected leg (if unilaterally affected). This part takes about 40 minutes to complete.

Part 3: The cool down part consists of walking and stretching exercises for the lower extremities for 10 minutes.

The circuit exercise program (part 2 above) is called a NEuroMuscular Exercise program (NEMEX)(5). The program has been tested feasible in severe hip and knee OA (6-8). The exercises are based on neuromuscular principles that aim at improving sensorimotor control and achievement of compensatory functional stability (also called dynamic stability) (5, 9, 10). Sensorimotor control is the ability to coordinate muscle activity and maintain functional stability during movement (10). Functional stability is the ability of the joint to remain stable during physical activity (9). Exercises in various positions (sitting, lying, standing) are used to achieve the desired postural activity.

When performing the neuromuscular exercise program the focus is on achieving good quality of the performance in each exercise with appropriate postural orientation and appropriate positioning of the joints in relation to each other, i.e., with the hip, knee and foot well aligned (Figure 1), thereby avoiding functional malalignment of the joints (Figure 2).

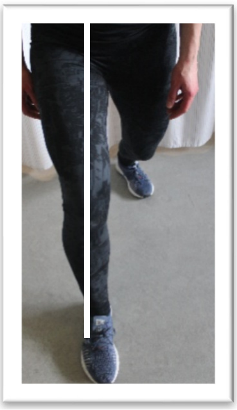

Figure 1
Proper alignment of the joints ie. that the hip, knee and foot are aligned/placed on a vertical line during the exercises.

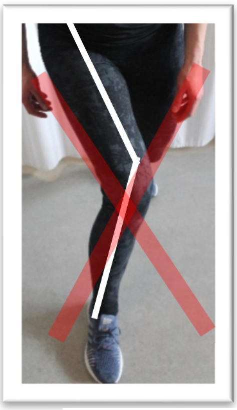

Figure 2
Functional malalignment of the joints during the exercises, which should be avoided.

Each of the participants receive individual introduction to each of the exercises where the start level for each exercise is chosen based on the execution and performance of the participants. The physiotherapist considers the participant prerequisites for performing the exercises. In this trial the physiotherapists supervise and motivate each of the participants individually using empowerment strategies.

To increase load/difficulty and facilitate improvement, the exercises can be progressed. The level and progression of each exercise is guided by the individual's neuromuscular control of the affected knee joint. Progression is made when an exercise is performed with good sensorimotor control and good quality of the performance (judged visually by the physiotherapist) and with minimal exertion and control of the movement (perceived by the patient).

Progression of the exercises are achieved by varying the number of repetitions, direction, velocity, load and/or the support surface. The exact means of progression depends on the focus of the specific exercise, i.e. whether the focus is on stability, postural orientation, postural function or strength. Four levels of difficulty are described for each exercise.

In the original GLAD program, the exercise part of the program can be either facility based, homebased or a combination. In this trial we have decided only to deliver the exercise program facility based in order to better record the adherence. This does not imply that the participants cannot perform additional exercises at home, but we neither encourage nor discourage them to do so. Nor do we record any homebased exercises.

For the purpose of this trial individual exercise diaries are filled-out by the participants and checked by the physiotherapist to ensure that the exercises are performed and progressed when relevant. The use of an exercise diary with detailed recording is an option but not mandatory according to the original GLAD description.

PAIN MANAGEMENT DURING EXERCISE

In this trial pain during and after each exercise session is assessed and recorded. A 0-10 numerical rating scale (NRS) is used, where 0 is “no pain” and 10 is “wort imaginable pain” (Figure 3) (11). A self-reported pain between 0-2 is considered “Safe”, pain between 3-5 is considered “Acceptable”, and pain from 6-10 is considered “High risk”. Participants are informed that pain up to 5 on this scale is allowed during and after a training session. The day after an exercise session the pain level should return to “pain as usual” defined as the self-reported pain level prior to exercise. If pain remains elevated, the level of training should be reduced at the next exercise session (5, 12).

In the original GLAD description, no explicit guidelines for management of pain >5 are provided. In this trial we have chosen that if a participant experiences knee pain intensity score >5 before commencing an exercise session, the warm-up is performed at low intensity at the ergometer bike followed by an examination by the physiotherapist. Based on a second pain assessment decisions are made on which exercises that are possible to perform that day. Likewise, a pain intensity score >5 during a specific exercise results in a modification/regression of the specific exercise that causes increased pain. This either by returning to the level before or reducing sets, reps, load, velocity, direction or modifying support surface. Furthermore, if unacceptable pain ratings (>5) persist for 3 consecutive exercise sessions, despite moderation of the exercises, the participant is referred to a medical doctor associated with the trial. All modifications of the exercises are registered in the exercise diary.

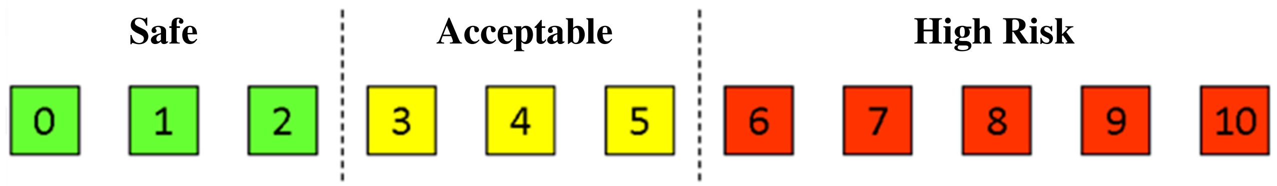

Figure 3: Visual Analog Scale for pain monitoring ranging from 0 (no pain) to 10 (pain as bad as it could be) with safe, acceptable, and risk pain zones indicated.

THE EXERCISE PROGRAM

<u>Part 1. Warm up</u>

Purpose: To enhance performance during the coming exercises and prevent injuries

Focus: To prepare the muscles and joints for the subsequent exercise via ergometer biking with individually adjusted load (Figure 4).

Time: 10 minutes

Progression: The workload is set individually and can be increased during the 10 minutes.

In this clinical trial the individual workload was set as the preferred level for each participant based on the instruction: you need to warm up to a level where you feel that you are prepared to exercise. If a participant has biked from the home to the gym the warm-up part may be skipped.

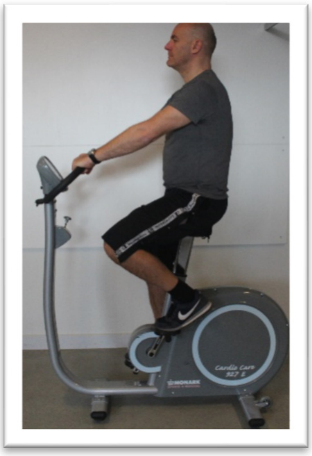

Figure 4. Warm up at ergometer bike

Part 2. Circuit program

The circuit program comprises of four exercise 'stations' focusing on: core stability/postural function; postural orientation; lower extremity muscle strength; and functional exercises. Several exercises are performed in each 'station'. Each participant performs the exercises at their individual level and uses approx. 40 minutes in total. Based on the level of each participant the physiotherapist will choose the appropriate start level for each of the exercises. The exercises can be performed in random order. The exercises are progressed as described below

Exercise station 1

Core stability

Purpose: This station includes exercises that focus on core/spine stability as this in theory affects a person's ability to stabilize and control his or her hip and knee joints during dynamic everyday movements.

Focus: Strengthening and neuromuscular control of the gluteus maximus, hamstrings, erector spinae and abdominal muscles.

Time: Not relevant, as the program focuses on repetitions.

Repetitions: 2-3 sets of 10-15 repetitions

Progression: The exercise is progressed from level 1 (easiest) to 4 (most difficult).

Progression is made when the participants can perform the exercise in sets of 3 with 15 repetitions with good sensorimotor control and good quality of the performance (based on visual inspection by the physiotherapist) and with minimal exertion and control of the movement (perceived by the patient).

Level 1 Core stability

A. Pelvic lift Lie on your back with your lower legs resting on top of the gym ball (Short lever arm). Arms down by your sides with the palms facing the floor. Lift your hips off the floor and push your hips toward the ceiling, then go back to the starting position. Avoid exaggerated lumbar curve (Figure 5).

B. Sit-ups Lie on your back, lower legs on top of the gym ball and arms stretched. Lift your head and upper back from the floor with the arms stretched and slightly lifted and lower yourself back down after a short pause (Figure 6).

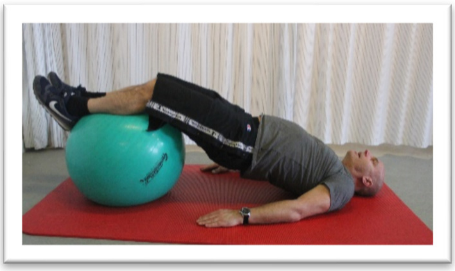

Figure 5. Pelvic lift, level 1

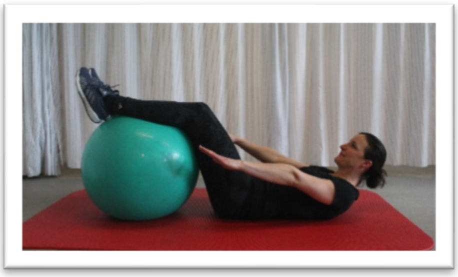

Figure 6. Sit-ups, level 1

Level 2 Core stability

A. Pelvic-lift Same as level 1, but with a longer lever arm so that only your heels are resting on top of the gym ball. Be careful not to overextend your knees when you lift your hips off the floor (Figure 7).

B. Sit-ups Lie on your back, lower legs on top of the gym ball and arms across your chest. Lift your head and upper back from the floor and lower yourself back down after a short pause (Figure 8).

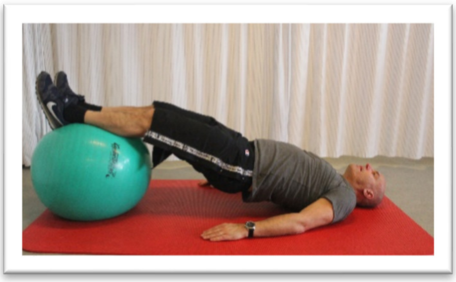

Figure 7. Pelvic lift, level 2

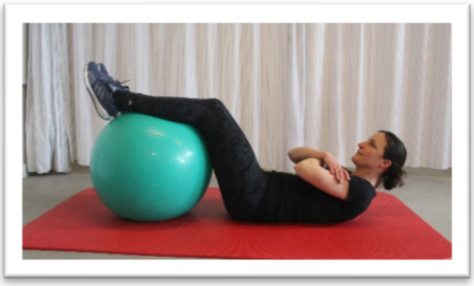

Figure 8. Sit-ups, level 2

Level 3 Core stability

A. Pelvic-lift Single leg pelvic lift. Be careful not to overextend your knee and control your pelvic tilt and rotation. Perform the exercise for both legs (Figure 9).

B. Sit-ups Lie on your back, lower legs on top of the gym ball and hands behind your neck. Lift your upper back from the floor and lower yourself back down after a short pause. Place your hands by your ears to avoid straining your neck (Figure 10).

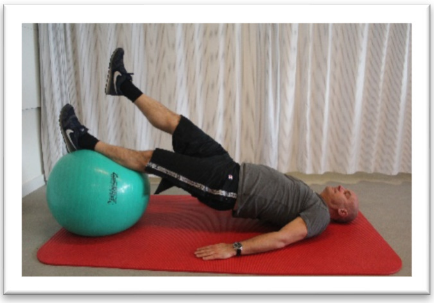

Figure 9. Pelvic lift, level 3

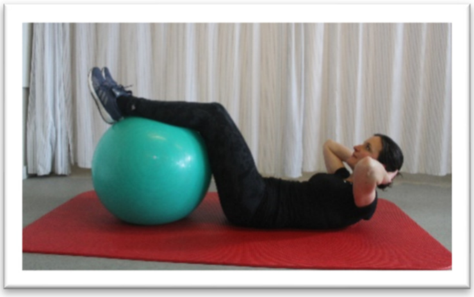

Figure 10. Sit-ups, level 3

Level 4 Core stability

A. Pelvic lift With your arms across your chest. The exercise can be done with one or both legs on the gym ball. Be careful not to overextend your knees and control your pelvic tilt and rotation (Figure 11).

B. Sit-ups Lie on your back, lower legs on top of the gym ball. while holding a pair of hand weights lift your upper back from the floor and lower yourself back down after a short pause (Figure 12).

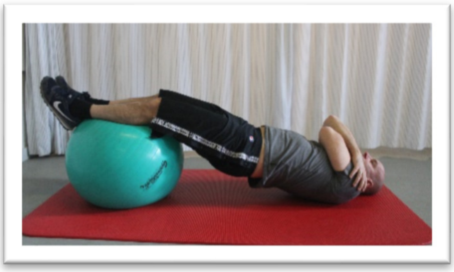

Figure 11. Pelvic lift, level 4

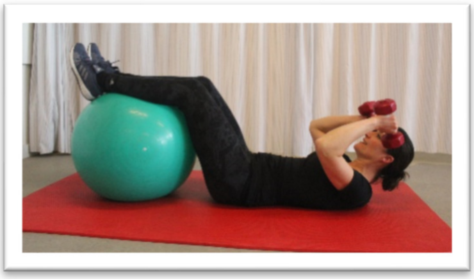

Figure 12. Sit-ups, level 4

Exercise station 2: Dynamic alignment of joints

Purpose: To increase neuromuscular control of the hip, knee and ankle joint

Focus: This station includes exercises that focus on alignment of the joints in relation to each other, i.e. that the hip, knee and ankle joints are properly aligned (Figure 1A).

Time: Not relevant, as the program focuses on repetitions.

Repetitions: 2-3 sets of 10-15 repetitions

Progression: The exercise is progressed from level 1 (easiest) to level 4 (most difficult).

Progression is made when the participants can perform the exercise in sets of 3 with 15 repetitions with good sensorimotor control and good quality of the performance (based on visual inspection by the physiotherapist) and with minimal exertion and control of the movement (perceived by the patient)

Level 1 Dynamic alignment of joints

A. Slide-exercise/step forward-backward: Standing position, weight-bearing on one leg, the other leg on a sliding surface. Slide backwards and forwards while bending the knee of the weight-bearing leg. Ensure proper alignment of hip, knee and ankle joints. Use hand support for balance if needed (Figure 13).

B. Slide-exercise/step sideways: Standing position, weight-bearing on one leg, other leg on sliding surface. Slide sideways while bending the knee of the weight-bearing leg, then slowly return to the starting position. Ensure proper alignment of hip, knee and ankle joints. Use hand support for balance if needed (Figure 14).

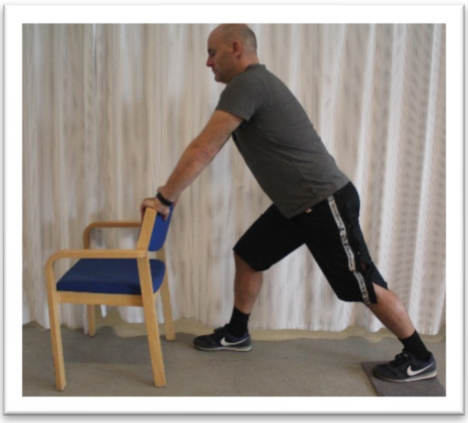

Figure 13. Slide-exercise/step forward-backward, level 1

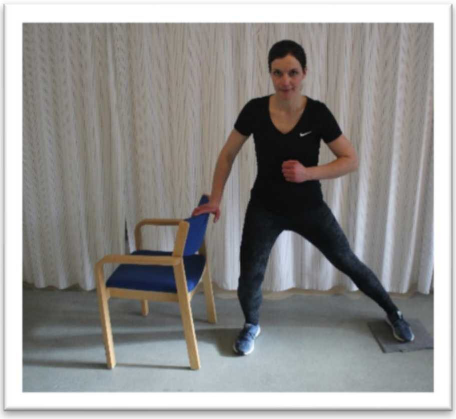

Figure 14. Slide-exercise/step sideways, level 1

Level 2 Alignment of joints

A. Slide-exercise/step forward-backward: Same as level 1 but standing on uneven surface with weight-bearing leg (e.g. foam pillow or thick mattress). Use hand support for balance if needed (Figure 15).

B. Slide-exercise/step sideways: Same as level 1 but standing on uneven surface with weight-bearing leg (e.g. foam pillow or thick mattress). Use hand support for balance if needed (Figure 16).

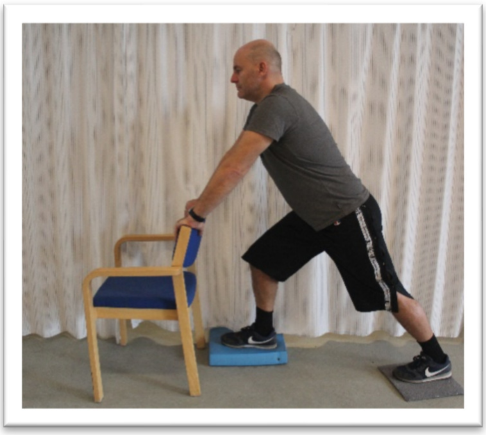

Figure 15. Slide-exercise/step forward-backward, level 2

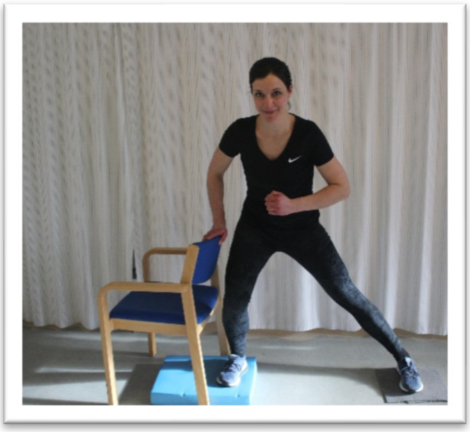

Figure 16. Slide-exercise/step sideways, level 2

Level 3 Dynamic alignment of joints

A. Forward lunge: Standing position, take a large step forward, bend the knee of the step-out leg while ensuring proper alignment of hip, knee and ankle joints, then push yourself back to the starting position (Figure 17).

B. Sideways lunge: Standing position, take a large step to the side, bend the knee of the step-out leg while ensuring proper alignment of hip, knee and ankle joints, then push yourself back to the starting position (Figure 18).

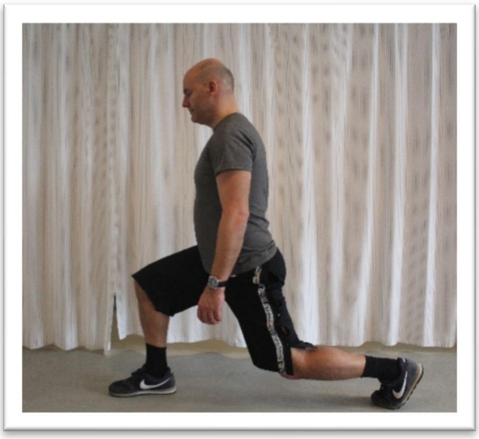

Figure 17. Slide-exercise/step forward-backward, level 3

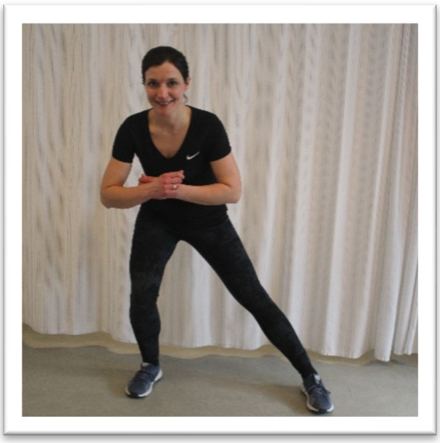

Figure 18. Slide-exercise/step sideways, level 3

Level 4 Dynamic alignment of joints

A. Forward lunge: Same as level 3 but holding hand weights (Figure 19).

B. Sideways lunge: Same as level 3 but with resistance band around ankles (Figure 20).

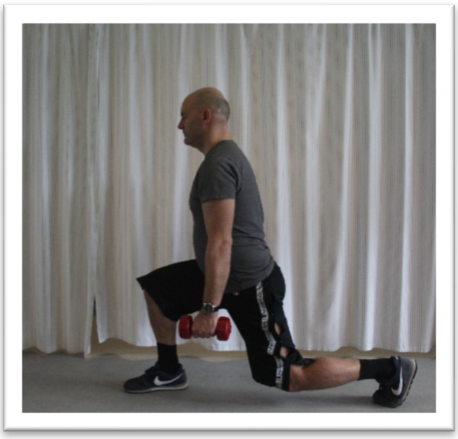

Figure 19. Slide-exercise/step forward-backward, level 4

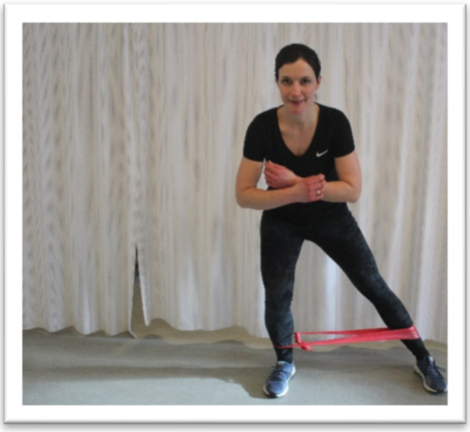

Figure 20. Slide-exercise/step sideways, level 4

Exercise station 3: Lower extremity muscle strength

Purpose: To strengthen the muscles controlling the movements; hip abduction, hip adduction, knee extension and knee flexion

Focus: The exercise should be done in a controlled movement, coming as close as possible to muscle failure. Therefore, choosing an elastic band that allows control as well as muscle fatigue is also of great importance.

Time: not relevant, as the program focuses on repetitions.

Repetitions: 2-3 sets of 10-15 repetitions

Progression: The exercises are progressed from level 1 (easiest) to 4 (most difficult).

Progression is made when the participants can perform the exercise in sets of 3 with 15 repetitions with good sensorimotor control and good quality of the performance (based on visual inspection by the physiotherapist) and with minimal exertion and control of the movement (perceived by the patient).

Level 1 Lower extremity muscle strength

A. Hip abductors: Standing with light resistance band in tension, lift your outer leg up and straight out to side as far as possible and stretch the band. Keep stance leg and torso stable, do not lean forward or to the side and do not twist your body. Be careful not to overextend the weight-bearing knee (Figure 21).

B. Hip adductors: Standing on one leg with light resistance band around the other leg. Pull your leg in towards the weight-bearing leg against the resistance of the band. Keep stance leg and torso stable, do not lean forward or to the side and do not twist your body. Be careful not to overextend the weight-bearing knee (Figure 22).

C. Knee extensors: Place the centre of a resistance band under one foot and hold an end in each hand. Bend and straighten your knee against the resistance of the band while keeping your hands still (Figure 23).

D. Knee flexors: Sitting position. Light resistance band around one foot and tied to something (e.g. a pole or a table) in front of you. Sit at the front of the chair to allow full range of movement when bending and straightening your knee. Pull the leg backwards against the resistance of the band and bend your knee (Figure 24).

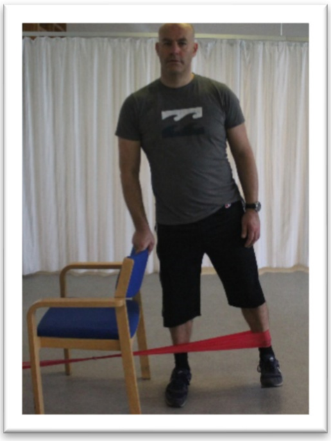

Figure 21. Hip abductors, level 1

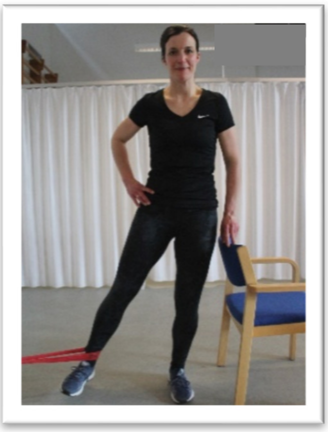

Figure 22. Hip adductors, level 1

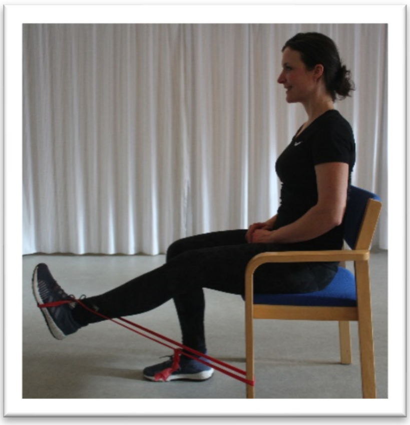

Figure 23. Knee extensors, level 1

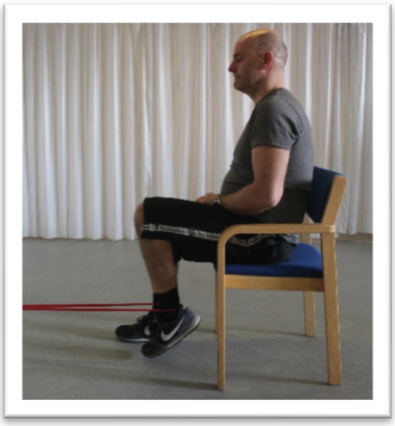

Figure 24. Knee flexors, level 1

Level 2 Lower extremity muscle strength

A. Hip abductors: Same as level 1, but with medium or heavy resistance band. (Figure 25).

B. Hip adductors: Same as level 1, but with medium or heavy resistance band (Figure 26).

C. Knee extensors: Sitting position with light resistance band tied to the chair and around one foot. Push resistance band forward by extending your knee (Figure 27).

D. Knee flexors: Same as level 1 but use medium resistance band (Figure 28).

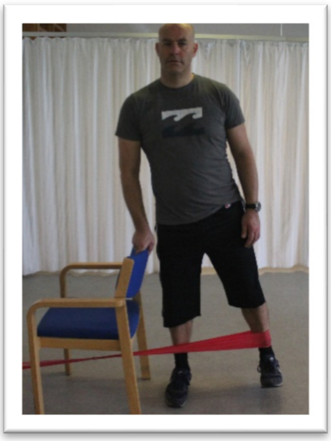

Figure 25. Hip abductors, level 2

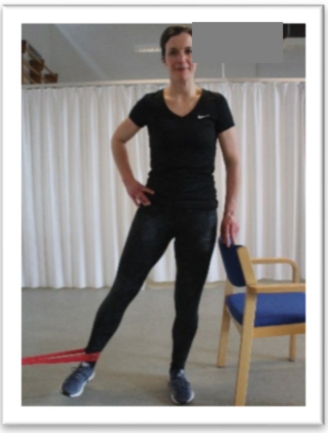

Figure 26. Hip adductors, level 2

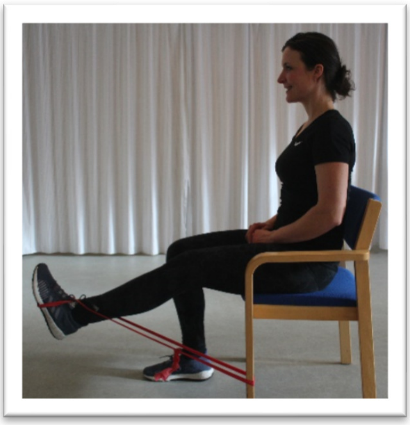

Figure 27. Knee extensors, level 2

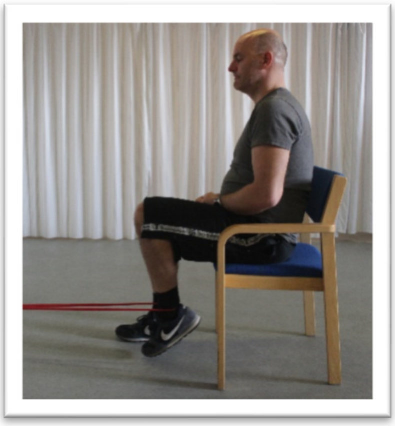

Figure 28. Knee flexors, level 2

Level 3 Lower extremity muscle strength

A. Hip abductors: Same as level 1-2 but standing on uneven surface (e.g. foam pillow or thick mattress) (Figure 29).

B. Hip adductors: Same as level 1-2 but standing on uneven surface (e.g. foam pillow or thick mattress) (Figure 30).

C Knee extensors: Same as level 2 but use medium-heavy resistance band (31).

D. Knee flexors: Same as level 1-2 but use heavy resistance band (32)

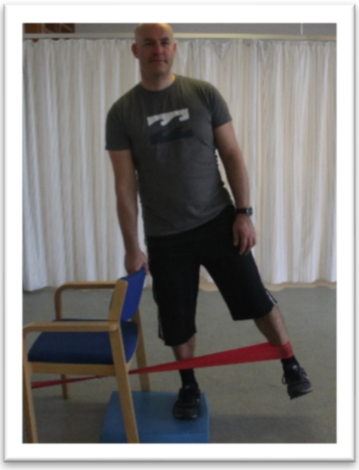

Figure 29. Hip abductors, level 3

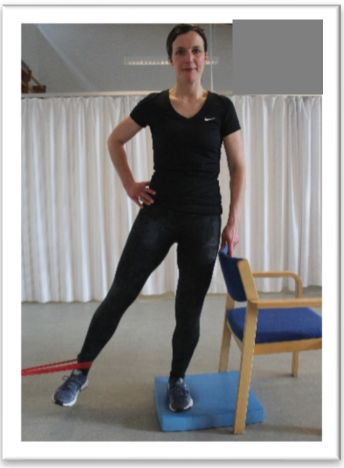

Figure 30. Hip adductors, level 3

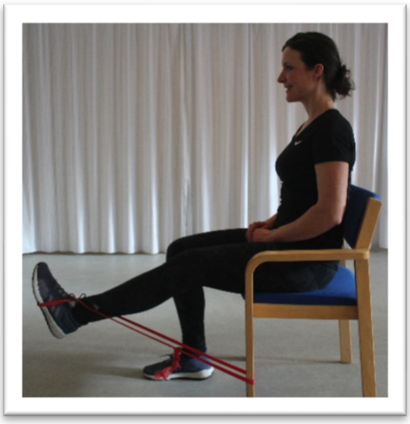

Figure 31. Knee extensors, level 3

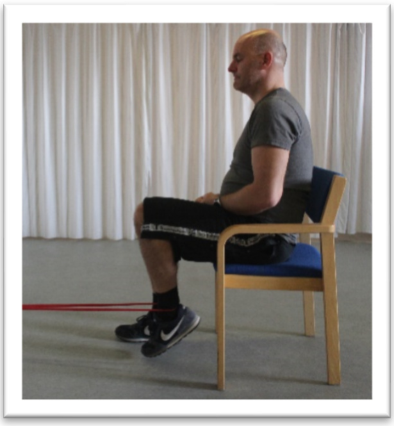

Figure 32. Knee flexors, level 3

Level 4 Lower extremity muscle strength

A. Hip abductors: Standing with a resistance band tied together in a circle around your ankles and weight-bearing on one leg. Lift your other leg up and straight out to side as far as possible while keeping stance leg and torso stable. To progress the exercise, try standing on a foam pillow (Figure 33).

B. Hip adductors: Lie down on one side, resting on your elbow and forearm. Place your upper leg on the chair while resting your lower leg on the floor. Raise the lower leg towards the bottom of the chair against gravity. Hold for a few seconds and return your leg to the floor. To progress the exercise, try moving further away from the chair so that only your foot is placed on the chair (Figure 34).

C. Knee extensors: Same as level 2-3 but use extra heavy resistance band (Figure 35).

D. Knee flexors: Same as level 1-3 but use extra heavy resistance band (Figure 36).

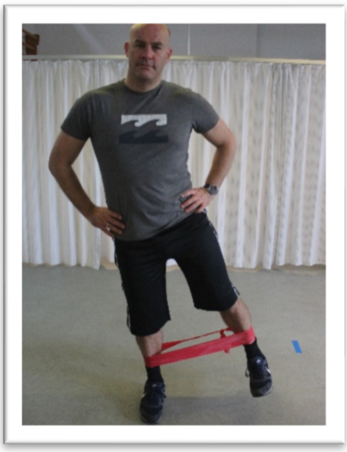

Figure 33. Hip abductors, level 4

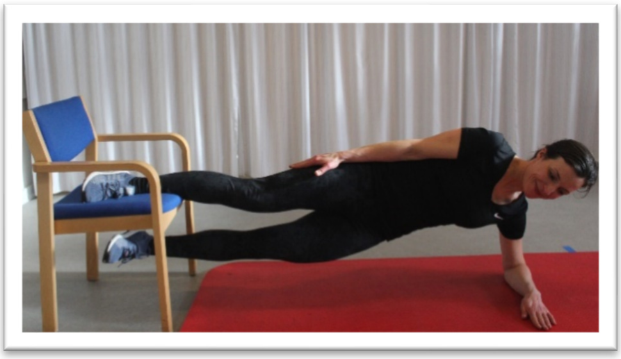

Figure 34. Hip adductors, level 4

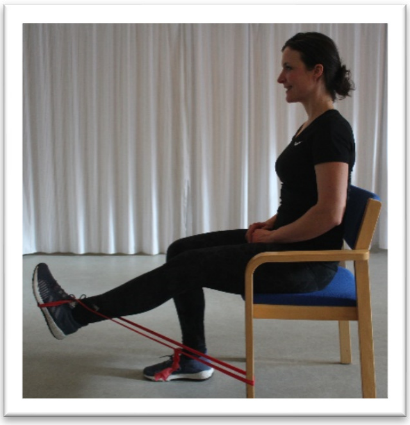

Figure 35. Knee extensors, level 4

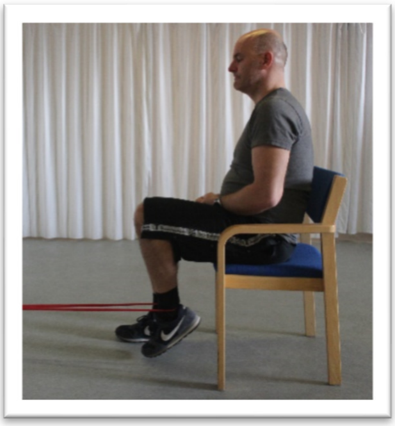

Figure 36. Knee flexors, level 4

Exercise station 4: Functional exercises

Purpose: To improve performance and prepare for daily activities.

Focus: Focus should be on controlling the body throughout the movement, to wean out bad movement patterns. Especially, the participants should focus on controlling their knees to stay in alignment with their hip and ankle and ensure to sit down on the chair in a controlled way.

Time: Not relevant, as the program focuses on repetitions.

Repetitions: 2-3 sets of 10-15 repetitions

Progression: The exercises are progressed from level 1 (easiest) to level 4 (most difficult).

Progression is made when the participants can perform the exercise in sets of 3 with 15 repetitions with good sensorimotor control and good quality of the performance (based on visual inspection by the physiotherapist) and with minimal exertion and control of the movement (perceived by the patient)

Level 1 Functional exercises

A. Chair stands: Start in a seated position, feet parallel and shoulder-width apart, putting load on both legs. Slight hand support for balance. Stand up while ensuring proper alignment of hip, knee and ankle joints. Do not let yourself collapse back down into the chair. Rather, control your lowering as much as possible (Figure 37).

B. Step ups: Step up onto a low step board or stair with or without slight hand support for balance. Then step backward to the starting position. Ensure proper alignment of hip, knee and ankle joints (Figure 38).

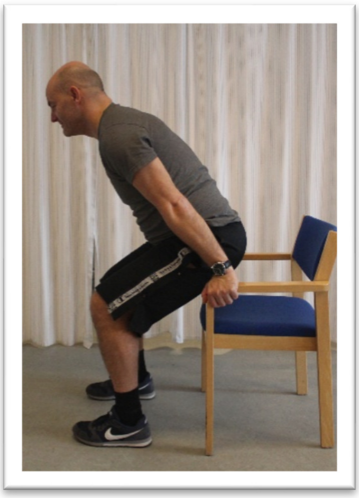

Figure 37. Chair stands, level 1

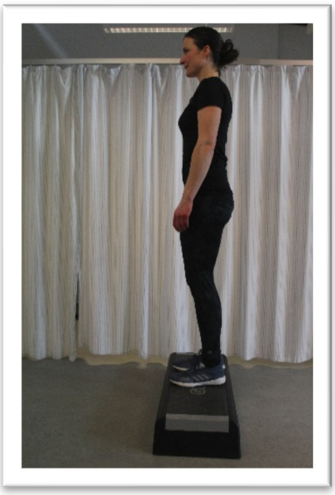

Figure 38. Step ups, level 1

Level 2 Functional exercises

A. Chair stands: Same as level 1, but without hand support (Figure 39).

B. Step ups: Same as level 1 but using a medium step board (Figure 40).

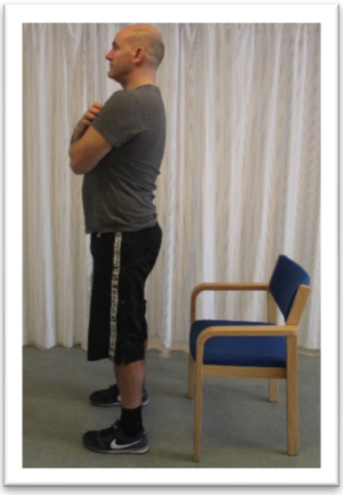

Figure 39. Chair stands, level 2

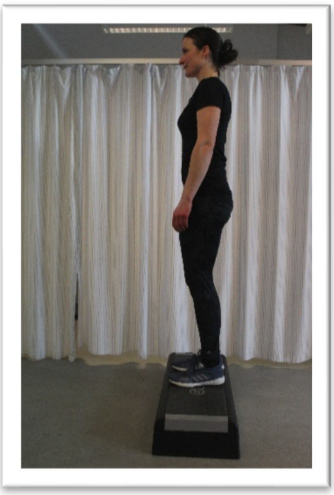

Figure 40. Step ups, level 2

Level 3 Functional exercises

A. Chair stands: Perform the exercise with one foot in front of the other. This exercise is a single leg stand up exercise with weight-bearing and focus on your back leg. Perform the exercise for both legs (Figure 41).

B. Step ups: Same as level 1-2 but using a high step board (Figure 42).

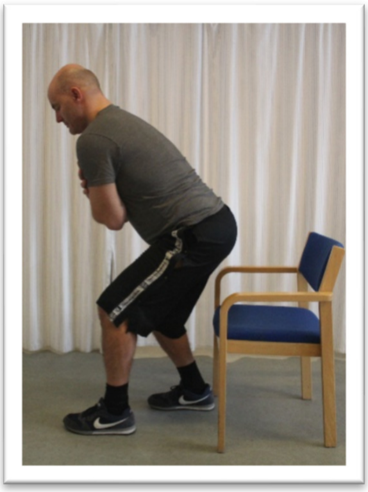

Figure 41. Chair stands, level 3

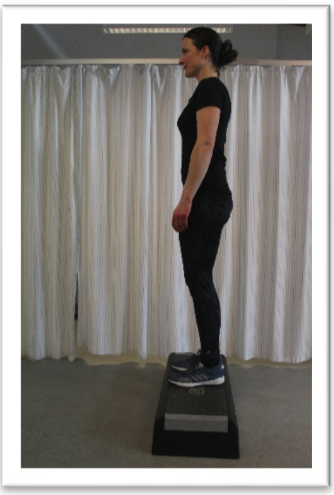

Figure 42. Step ups, level 3

Level 4 Functional exercises

A. Chair stands: Perform the exercise same as level 2 while holding hand weights. Keep your elbows at a 90-degree angle (Figure 43).

B. Step ups: Step up onto the step board with one leg and step across and back again with your other leg (Figure 44)

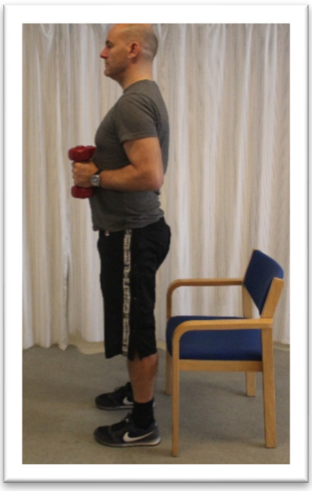

Figure 43. Chair stands, level 4

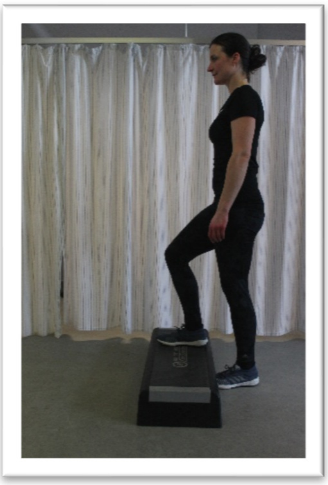

Figure 44. Step ups, level 4

Part 3. Cooling down

Purpose: To increase mobility and flexibility of muscles and tendons and to minimise muscle soreness and cramps.

Focus: Participants should keep stretches for at least 30 seconds before letting go. Walking should be done at a not-exhausting level.

Time: It is up to the participant how much time they want to spend on cooling down

Repetitions: It is up to the participant how many times they want to stretch or walk.

Walking exercise

Walk forwards and backwards in front of a mirror with focus on proper alignment of the hip, knee and ankle joints (Figure 45).

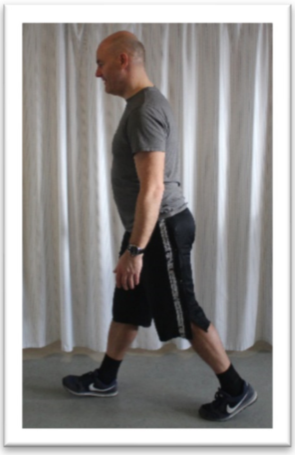

Figure 45. Walking forwards and backwards

Stretching

Stretching of the major muscle groups of the lower extremities will increase flexibility. Hold the stretch for at least 30 seconds. For added support, stand by a wall or something to support yourself while doing the stretches. Focus on appropriate alignment of the hip, knee and ankle joints (Figure 46 A-D)

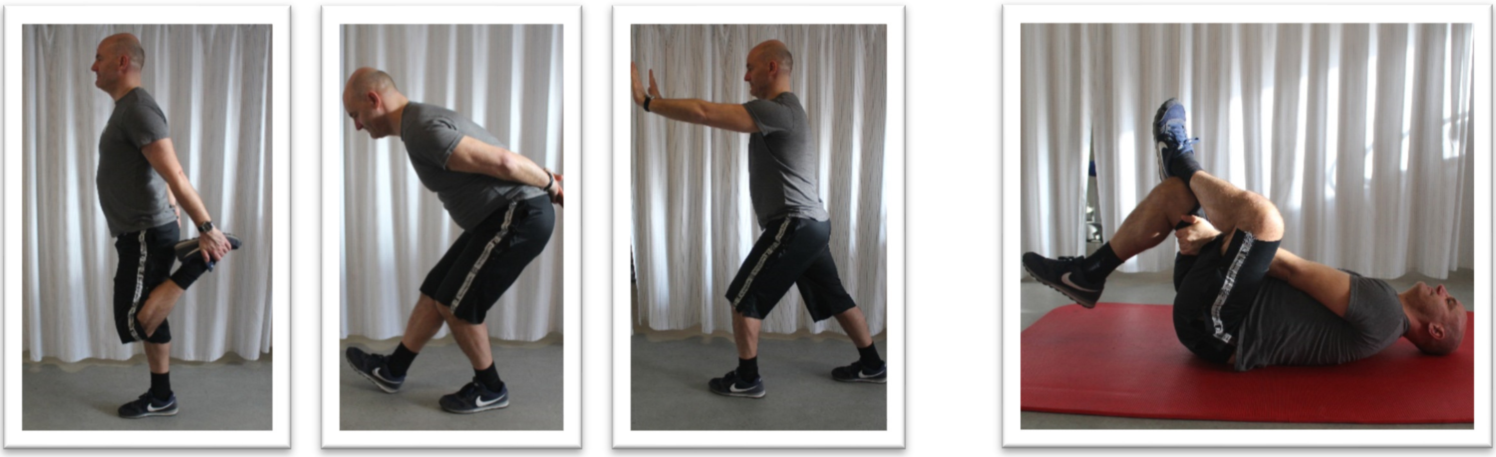

Figure 46 A-D. Stretches of the lower extremities

In this clinical trial participants are introduced by the physiotherapists to the cooling down part of the program. The participants individually choose if they want to perform this. The cooling down part is not registered in the diary.

References

1. Skou ST, Roos EM. Good Life with osteoArthritis in Denmark (GLA:D): evidence-based education and supervised neuromuscular exercise delivered by certified physiotherapists nationwide. BMC Musculoskelet Disord. 2017;18(1):72.
2. Slade SC, Dionne CE, Underwood M, Buchbinder R. Consensus on Exercise Reporting Template (CERT): Explanation and Elaboration Statement. Br J Sports Med. 2016;50(23):1428-37.
3. Hoffmann TC, Glasziou PP, Boutron I, Milne R, Perera R, Moher D, et al. Better reporting of interventions: template for intervention description and replication (TIDieR) checklist and guide. BMJ. 2014;348:g1687.
4. Sundhedsstyrelsen. Knæartrose - nationale kliniske retningslinjer og faglige visitationsretningslinjer https://www.sst.dk/da/udgivelser/2012/nkr-knaeartrose: Sundhedsstyrelsen; 2012 [
5. Ageberg E, Link A, Roos EM. Feasibility of neuromuscular training in patients with severe hip or knee OA: the individualized goal-based NEMEX-TJR training program. BMC Musculoskelet Disord. 2010;11:126.
6. Skou ST, Odgaard A, Rasmussen JO, Roos EM. Group education and exercise is feasible in knee and hip osteoarthritis. Dan Med J. 2012;59(12):A4554.
7. Ageberg E, Nilsdotter A, Kosek E, Roos EM. Effects of neuromuscular training (NEMEX-TJR) on patient-reported outcomes and physical function in severe primary hip or knee osteoarthritis: a controlled before-and-after study. BMC Musculoskelet Disord. 2013;14:232.
8. Villadsen A, Overgaard S, Holsgaard-Larsen A, Christensen R, Roos EM. Immediate efficacy of neuromuscular exercise in patients with severe osteoarthritis of the hip or knee: a secondary analysis from a randomized controlled trial. J Rheumatol. 2014;41(7):1385-94.
9. Ageberg E, Roos EM. Neuromuscular exercise as treatment of degenerative knee disease. Exerc Sport Sci Rev. 2015;43(1):14-22.
10. Williams GN, Chmielewski T, Rudolph K, Buchanan TS, Snyder-Mackler L. Dynamic knee stability: current theory and implications for clinicians and scientists. J Orthop Sports Phys Ther. 2001;31(10):546-66.
11. Thomee R. A comprehensive treatment approach for patellofemoral pain syndrome in young women. Phys Ther. 1997;77(12):1690-703.
12. Bartholdy C, Klokker L, Bandak E, Bliddal H, Henriksen M. A Standardized "Rescue" Exercise Program for Symptomatic Flare-up of Knee Osteoarthritis: Description and Safety Considerations. J Orthop Sports Phys Ther. 2016;46(11):942-6.
